# Saffron Samples of Different Origin: An NMR Study of Microwave-Assisted Extracts

**DOI:** 10.3390/foods3030403

**Published:** 2014-07-08

**Authors:** Anatoly P. Sobolev, Simone Carradori, Donatella Capitani, Silvia Vista, Agata Trella, Federico Marini, Luisa Mannina

**Affiliations:** 1 Institute of Chemical Methodologies, Magnetic Resonance Laboratory “Annalaura Segre”, National Research Council (CNR), Via Salaria km 29.300, 00015 Monterotondo, Rome, Italy; E-Mails: anatoli.sobolev@imc.cnr.it (A.P.S.); donatella.capitani@cnr.it (D.C.); 2 Department of Drug Chemistry and Technologies, Sapienza University of Rome, P.le Aldo Moro 5, 00185 Rome, Italy; E-Mails: simone.carradori@uniroma1.it (S.C.); vistasilvia@gmail.com (S.V.); 3 Department of Chemistry, Sapienza University of Rome, P.le Aldo Moro 5, 00185 Rome, Italy; E-Mails: agata.trella@gmail.com (A.T.); federico.marini@uniroma1.it (F.M.)

**Keywords:** saffron, NMR, metabolic profiling, microwave-assisted extraction

## Abstract

An NMR analytical protocol is proposed to characterize saffron samples of different geographical origin (Greece, Spain, Hungary, Turkey and Italy). A microwave-assisted extraction procedure was developed to obtain a comparable recovery of metabolites with respect to the ISO specifications, reducing the solvent volume and the extraction time needed. Metabolite profiles of geographically different saffron extracts were compared showing significant differences in the content of some metabolites.

## 1. Introduction

**Figure 1:**
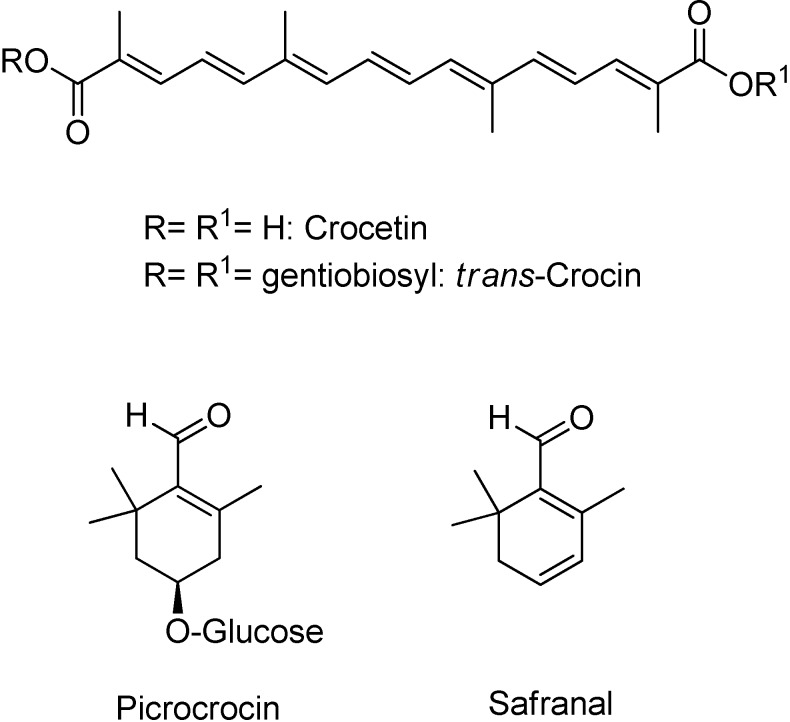
The chemical structure of the main bioactive compounds from the dried stigmas of *Crocus sativus* L.

Saffron is a very expensive spice cultivated in few countries (Iran, India, Spain, Greece, Italy and Morocco), and it is mainly used as a food additive and for coloring purposes. The spice is obtained from the dried stigmas of the plant, *Crocus sativus* L. (Iridaceae). It has recently gained interest as a potential source of pharmacologically bioactive compounds, such as crocetin, picrocrocin and safranal, see Figure 1 [1,2,3,4,5,6]. This finding makes the spice a promising candidate for being a functional food. A functional food is considered a natural or processed food that contains known biologically active metabolites in defined quali-quantitative amounts providing a clinically proven health benefit. However, different drying procedures and post-harvest techniques, based on cultural and geographical traditions, lead to different metabolite patterns and concentrations of its constituents [7]. As a result of this, several analytical techniques have been proposed to discriminate the geographical origin on the basis of the chemical composition of this spice [8,9,10,11,12]. Moreover, its limited production and high price have markedly favored both a large number of commercial adulterations and the concurrent development of strict normative certifying of saffron’s authenticity and quality [13].

Saffron contains several main metabolites analyzed by different methods and instruments, because of their distinct chemical-physical profile. The presence of these compounds is important to achieve the recognized sensorial properties of the spice (color, taste and aroma), and all of them have been tested for potential therapeutic applications. Crocins are a group of glycosides (mono-glycosyl or di- glycosyl esters) of a C20 carotenoid aglycone crocetin (Figure 1). The most abundant glycoside is the crocetin digentiobiosyl ester (all *trans*-crocin), completely water-soluble compared to its parent compound. Crocetin and its esters are routinely analyzed by HPLC/UV-Vis, Raman spectroscopy and a non-aqueous capillary electrophoresis method [14]. The main component responsible for the bitter taste of saffron is picrocrocin, a quite polar terpenoid glycoside, present in an amount of 1%–13% in the stigmas. The characteristic odor of saffron is attributed to safranal (the main component of the volatile fraction and the product of natural deglycosylation of picrocrocin) and to other carotenoid breakdown products with a similar structure, typically determined by GC or GC/MS [15].

Techniques reported in the literature for the isolation of specific bioactive compounds, reported in Figure 1 (targeted analysis), differ from each other on the basis of the chemical-physical characteristics (*i.e.*, molecular weight, polarity, water solubility, volatility) of the metabolite considered. In general, conventional extraction with solvents and ultrasound-assisted extraction have been preferred for polar compounds, whereas steam distillation and supercritical fluid extraction have been chosen for volatile components. The extraction is, therefore, one of the most crucial steps for the complete recovery of all valuable plant secondary metabolites. To the best of our knowledge, automatic microwave-assisted extraction has never been applied to *Crocus sativus* L. stigmas and its well-known advantages (reduced solvent volume and extraction time, pressurized and sealed vials to reduce loss of volatile components, strict control of multiple parameters, such as temperature, pressure and irradiation power) make it an interesting method to achieve an exhaustive extract of this plant [16]. Finally, this technique provides, in general, a better isolation of secondary metabolites, due to the complete swelling of the subcellular structures under microwave irradiation, which leads to a deeper penetration of the solvent and an easier release of intracellular metabolites.

NMR methodology is one of the most suitable methodologies to obtain ‘‘high-throughput’’ spectroscopic/structural information on a wide range of compounds with a high analytical precision. It has been applied to the analysis of food following different approaches, that is target analysis, metabolic profiling and metabolic fingerprinting [17]. Several foodstuffs have been investigated to obtain information on varieties, geographical origin, quality, adulteration, and so on [18,19,20,21,22,23].

Yilmaz *el al*. [12] have used the ^1^H NMR metabolic fingerprinting approach, which does not require a detailed spectral assignment, to assess the quality of Iranian saffron using also an unsupervised classification method to detect adulteration.

Here, the ^1^H-based metabolic profiling of microwave-assisted saffron extracts is reported. The assignment of some compounds is reported in more detail with respect to literature data [12,24,25,26]. A simple and environmentally-friendly microwave-assisted extraction (MAE) requiring only a minimal amount of these expensive dried stigmas is proposed to obtain the major number of metabolites. Finally, a comparison of the ^1^H metabolic profiling of saffron samples of different geographical origin was carried out.

## 2. Experimental Section

### 2.1. Materials

Deuterated solvent (CD_3_OD, 99.8 atom% of deuterium) was purchased from Euriso-Top (Saclay, France). Saffron samples (300 mg) from Greece, Spain, Turkey, Italy (three different regions) and Hungary (see Table 1 for details) belonging to the same harvest (2012) were purchased from local producers and markets and kept at 4 °C in the dark until the analysis.

**Table 1:** Saffron samples used in the study.

Sample Code	Country (region)	Producer
AB	Italy (Abruzzi) PDO*	Agricultural company “Matergia”, Barisciano (Aquila)
GR	Greece	Local market
LA	Italy (Latium)	Agricultural company “Roncaglia”, Ronciglione (Viterbo)
SA1	Italy (Sardinia)	Local market
SA2	Italy (Sardinia)	“Zafferano Monreale snc” San Gavino Monreale, (Cagliari)
SA3	Italy (Sardinia)	Agricultural company “Curreli Franco” San Gavino Monreale, (Cagliari)
SA4	Italy (Sardinia) PDO	Agricultural company “Itria”, Turri (VS)
SP1	Spain	“Don Jerez”
SP2	Spain	“Cameo Zafferano Red”
TK	Turkey	Local market
HU	Hungary	“Nehéz Gyula”

* PDO, Protected Designation of Origin.

### 2.2. Microwave-Assisted and Conventional Extractions

All dried samples were ground manually in a mortar and sifted to obtain a more uniform granulometry before performing the microwave-assisted extraction.

Microwave-assisted extraction (MAE) was performed by an automatic Biotage Initiator™ 2.0 (Uppsala, Sweden; 2.45 GHz high-frequency microwaves; power range: 0–300 W). The internal vial temperature was controlled by an IR sensor probe. Manually-ground saffron (10 mg) was placed in a 10-mL sealed vessel suitable for an automatic single-mode microwave reactor, and 0.8 mL of CD_3_OD were added to the sample to form a yellow-orange suspension. The sample was heated by microwave irradiation for 10, 19 and 30 min at 40 °C, followed by cooling with pressurized air. An internal standard (3-(trimethylsilyl)-propionic-2,2,3,3-*d*_4_ acid sodium salt, TSP, 1 mM) was added, and the suspension was stirred magnetically for 1 min in the dark. The liquid phase was carefully separated from the solid precipitate for the NMR analysis.

For comparison, conventional extraction was performed with 10 mg of manually-ground saffron placed in a 10-mL sealed vessel and 0.8 mL of CD_3_OD to form a yellow-orange suspension. The sample was magnetically stirred for 30 min in the dark at room temperature. Then, the internal standard (TSP, 1 mM) was added, and the suspension was magnetically stirred for 1 min in the dark. The liquid phase was carefully separated from the solid precipitate for the NMR analysis.

### 2.3. High-Resolution NMR Measurements

The NMR spectra of saffron samples were recorded in CD_3_OD at 27 °C on a Bruker AVANCE 600 NMR spectrometer operating at a proton frequency of 600.13 MHz and equipped with a Bruker multinuclear z-gradient inverse probe head capable of producing gradients in the z-direction with a strength of 55 G cm^−1^. ^1^H spectra were referenced to the methyl signal of TSP at 0.00 ppm, whereas ^13^C spectra were referenced to the CD_3_ resonance of deuterated methanol (*δ* = 49.0 ppm).

^1^H spectra of methanol extracts were acquired by coadding 128 transients with a recycle delay of 9 s. The experiment was carried out by using a 45° pulse, 32 K data points. Presaturation of the HDO signal with a soft pulse during 2 s just before acquisition was applied using a modified zgpr Bruker pulse sequence.

2D NMR experiments, namely ^1^H-^1^H TOCSY, ^1^H-^13^C heteronuclear single quantum coherence (HSQC) and ^1^H-^13^C heteronuclear multiple-bond correlation (HMBC) [27] were performed using the same experimental conditions previously reported [28]. The mixing time for the ^1^H-^1^H TOCSY was 80 ms. The HSQC experiments were performed using a coupling constant ^1^*J*_C–H_ of 150 Hz, and the ^1^H-^13^C HMBC experiments were performed using a delay for the evolution of long-range couplings of 80 ms.

The diffusion ordered spectroscopy (DOSY) experiment [29] was performed using double stimulated echo and LED (dstegp3s pulse sequence in Bruker pulse library) with a sine-shaped gradient of different intensities. The gradient strength was logarithmically incremented in 32 steps from 2% up to 95% of the maximum gradient strength (55 G cm^−1^). The following experimental settings were used: diffusion time, Δ, was 100 ms; gradient duration, δ, was 2.6 ms; the longitudinal eddy current delay was 25 ms; the gradient pulse recovery time was set to 0.1 ms. After Fourier transformation and baseline correction, the diffusion dimension was processed by means of the Bruker TOPSPIN software (version 1.3).

### 2.4. Measurement of the Metabolic Content and Statistical Analysis

Using the Bruker TOPSPIN 1.3 software and defined integral regions, the integrals of selected ^1^H resonances were measured with respect to the integral of TSP CH_3_ signal at 0.00 ppm normalized to 100 and used as the internal standard. The measured resonances (see Table 2) are due to fatty acids (FA) (0.90 ppm), acetic acid (AcOH) (1.91 ppm), picrocrocin (PCROC) (2.15 ppm), linoleic fatty acid (C18:2) (2.78 ppm), linolenic fatty acid (C18:3) (2.81 ppm), β-glucose (βGLC) (3.12 ppm), α-glucose (αGLC) (5.10 ppm), all double bonds in fatty acids (DB) (5.34 ppm), β-d-gentiobiosyl moiety in crocins (GB-CROC) (5.54 ppm), β-d-glucosyl moiety in crocins (βGLC-CROC) (5.56 ppm), all-*trans*- crocetin moiety in crocins (DBtCROC) (7.44 ppm) and 13-*cis*-crocetin moiety in crocins (DBcCROC) (7.51 ppm).

The statistical processing of NMR data was performed using the STATISTICA package for Windows (version 5.1, 1997) and by in-house routines written in MATLAB (The Mathworks, Natick, MA, USA). Before performing the statistical analysis, the selected variables were mean-centered, and each variable was divided by its standard deviation (autoscaling). Principal component analysis (PCA) was performed on the 12 selected variables: the percentage of variance for each specific principal component is reported. PCA results are shown reporting the scores of principal components and also as a plot of variable loadings.

## 3. Results and Discussion

### 3.1. Microwave-Assisted Extraction

Saffron stigmas contain metabolites of a wide polarity spectrum. For instance, picrocrocin and crocins are glycosides and, therefore, dissolve excellently in water, whereas aromatic compounds and lipids are immiscible in water, but soluble in organic solvents. Methanol has been suggested as a good compromise to obtain an untargeted metabolic profiling, including polar, as well as non-polar constituents [12].

It was preliminary found that the metabolite concentration *vs*. extraction time followed a saturation curve. Plant material visibly lost the natural color after 30 min, and the 30-min extract showed a major amount of metabolites with respect to the 10- and 19-min extracts, respectively, according to the NMR profiling spectra.

In order to study the influence of temperature on the extraction process and on the product recovery and stability, a comparison between the conventional and the microwave-assisted extraction (MAE) technique was performed. A temperature of 40 °C (MAE) did not enhance metabolite degradation and was found to be as efficient as the conventional extraction at room temperature (by means of a direct comparison of their NMR metabolite profiling). Better results were achieved with respect to the reported ISO specifications regarding the limited volume of the extraction solvent for the analysis (0.8 mL of methanol), the reduced time of extraction (30 min *vs.* 60 min) and the possibility to work with small amounts (10 mg) of this expensive spice. In addition, the microwave-assisted extraction directly provides the sample for the NMR analysis without any further pre-treatment, avoiding the loss and/or degradation of natural constituents (*i.e.*, volatile fraction) and enhancing the automation of the process.

### 3.2. NMR Analysis of Saffron Extracts

**Figure 2:**
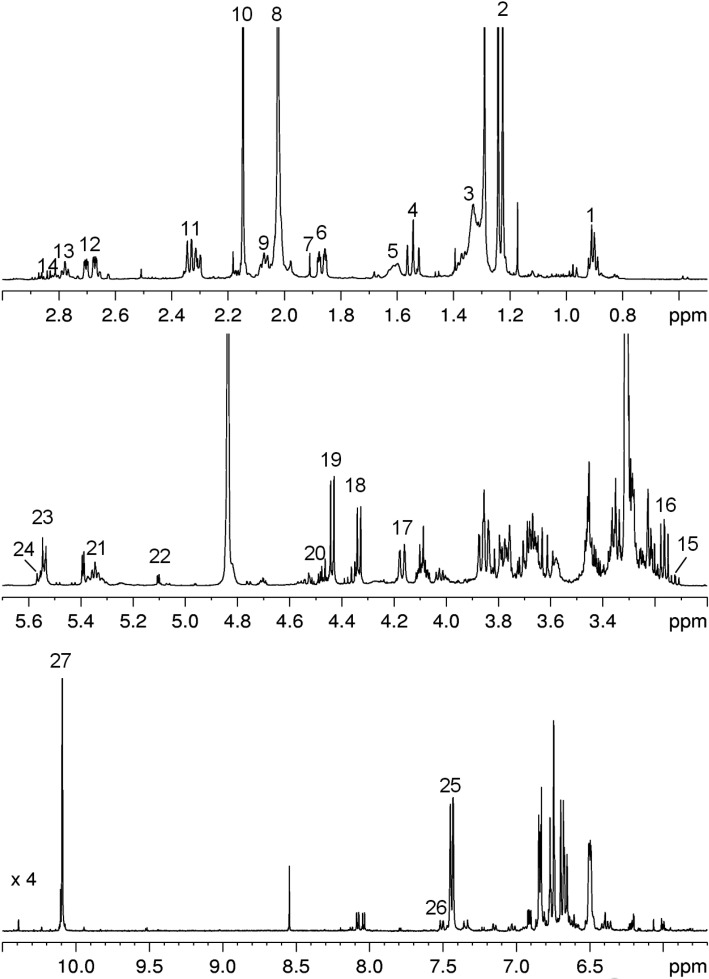
^1^H NMR spectrum of saffron extract in CD_3_OD at 27°C. Assignments: 1, CH_3_ FA; 2, CH_3_-7,8 PCROC (picrocrocin); 3, CH_2_ FA; 4, CH_2_-5PCROC; 5, CH_2_-3 FA; 6, CH_2_-5 PCROC; 7, CH_3_ AcOH; 8, CH_3_-19,20 crocetin; 9, CH_2_-2 FA; 10, CH_3_-9 PCROC; 11 and 12, CH_2_-3 PCROC; 13, CH_2_-11 C18:2; 14, CH_2_-11 C18:3; 15, CH-2 β-glucose (βGLC); 16, CH-2′ PCROC; 17, CH_2_-6 GB-CROC; 18, CH-1′ GB-CROC; 19, CH-1′ βGLC-CROC; 20, CH-1 β-glucose; 21, DB (double bond); 22, CH-1 α-glucose; 23, CH-1 GB-CROC; 24, CH-1 βGLC-CROC; 25, CH-10,10′ all-*trans*-crocin; 26, CH-10 13-*cis*-crocin; 27, CHO-10 PCROC.

The ^1^H spectrum of microwave-assisted saffron extract is reported in Figure 2. The ^1^H and ^13^C assignments were obtained by literature data [12,24,25,26,30] and 1D and 2D NMR experiments; see Table 2. In particular, the assignment of ^13^C signals from the all-*trans*-crocin (sugar and aglycone moieties) and picrocrocin was obtained directly in the CD_3_OD extract using the ^1^H-^13^C HSQC experiment, whereas it was previously reported only in the case of isolated compounds and in other solvents.

**Figure 3:**
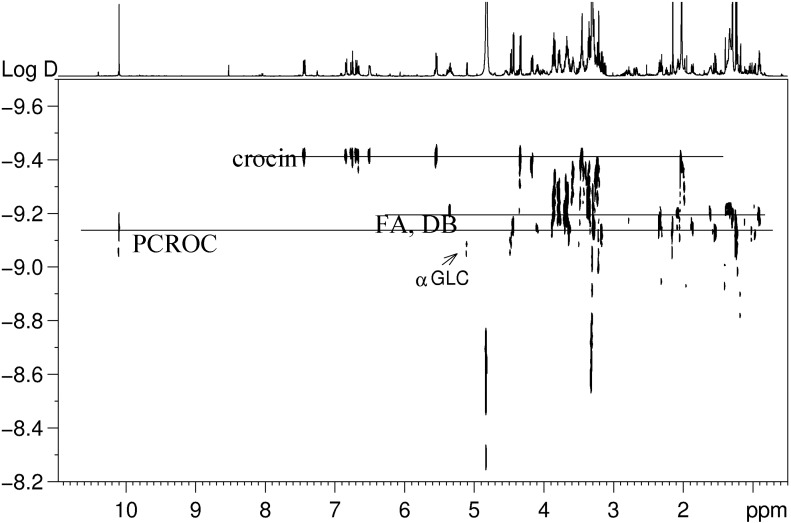
2D DOSY map of saffron extract in CD_3_OD at 27°C. All signals from a given molecule show the same diffusion coefficient as indicated for the case of crocin, fatty cids and picrocrocin.The ^1^H spectrum of the extract is also reported as a horizontal projection.

The ^1^H spectrum shows not only signals of crocin and picrocrocin typical of saffron, but also signals of other compounds, such as lipids and sugars. In particular, linolenic and linoleic fatty acids and phosphatidylcholine were detected together with glucose present in α and β forms. Interesting information on the saffron extract can be obtained analyzing the DOSY map reported in Figure 3. DOSY is a particularly convenient way of displaying the molecular self-diffusion information in a bi- dimensional array, with the NMR spectrum in one dimension and the self-diffusion coefficient in the other one [29]. As expected for a mixture, also in the case of the saffron extract, many signals with different self-diffusion coefficients and, therefore, with different molecular weights are present, due to different metabolites; on the other hand, signals from the same molecule show the same self-diffusion coefficient. The DOSY map shows clearly the presence of all-*trans*-crocin, having the same diffusion coefficients for the aglycone moiety and sugars (glucose and gentiobiose), whereas crocetin seems not to be present. Picrocrocin was also observable, the aldehydic signal and the sugars signals having the same diffusion coefficient. Finally, free glucose with the highest self-diffusion coefficient value was easily detected.

**Table 2:** Summary of the metabolites identified in the 600 MHz ^1^H spectrum of the microwave-assisted extract of saffron. * Indicate the signals selected for statistical analysis.

Compound	Assignment	¹H, ppm	Multiplicity, *J*_H–H_, Hz	¹³C, ppm
*Linoleic acid* C18:2	CH_2_-2	2.33		
	CH_2_-3	1.61		
	CH_2_-4	1.34		
	CH_2_-8,14	2.07	t: 7.4	
	CH-9,10,12,13	5.34 *		
	CH_2_-11	2.78 *	t: 6.8	
	CH_2_-15,16,17	1.32		
	CH_3_-18	0.91	t: 7.0	
*Linolenic acid* C18:3	CH_2_-1,4,5,6,7	1.34		
	CH_2_-2	2.33		
	CH_2_-3	1.61		
	CH_2_-8	2.08	t: 7.4	
	CH-9,10,12,13	5.34		
	CH_2_-11,14	2.81 *	t: 6.8	
	CH-15	5.30 *		
	CH-16	5.38 *		
	CH_2_-17	2.08	t: 7.4	
	CH_3_-18	0.97	t: 7.0	
*Phosphatidylcholine*	(CH_3_)_3_N	3.21	s	54.6
*Acetic acid* AcOH	CH_3_	1.91 *	s	
*All fatty acids except* C18:3, FA	CH_3_	0.90 *	t: 6.7	14.6–14.4
*All*-trans-*crocin* DBtCROC (aglycone)	C-8,8′			168.6
	C-9,9′			126.5
	CH-10,10′	7.44 *	dm: 11.3	141.9
	CH-11,11′	6.68	dd: 15.0; 11.4	124.7
	CH-12,12′	6.76	d: 15.0	146.2
	C-13,13′			138.1
	CH-14,14′	6.50	dm: 7.7	137.4
	CH-15,15′	6.84	dd: 7.8; 2.9	133.0
	CH3-19,20	2.02	s	12.6; 12.9
*13*-cis-*crocin* DBcCROC (aglycone)	CH-10	7.51 *	d: 11.8	
	CH-10′	7.44		
	CH-11,11′	6.68		
	CH-12	7.34	d: 14.8	
	CH-12′	6.76		
	CH-14	6.37	d: 12.2	
	CH-14′	6.50		
	CH-15	7.03	dd: 13.4; 12.8	
	CH-15′	6.74		
(β-d-gentiobiosyl) (GB-CROC)	CH-1	5.54 *	d: 7.7	95.9
	CH-2	3.45		77.8
	CH-3,4,5	3.56–3.39		
	CH_2_-6	4.17	dd: 11.5; 2	69.5
		3.78		69.5
	CH-1′	4.33	d: 7.8	104.5
	CH-2′	3.23		75.1
	CH-3′	3.34		
	CH_2_-6′,6′	3.85	m	62.7
		3.66	m	62.7
(β-d-glucosyl)	CH-1	5.56 *	d: 7.7	96.0
(βGLC-CROC)	CH_2_-6, 6	3.84; 3.69		62.7
*Picrocrocin* (PCROC) (aglycone)	C-1			141.1
	C-2			155.6
	CH_2_-3,3	2.69	ddd: 18.7; 5.5; 2.2	42.4
		2.31	ddm: 18.7; 9	42.4
	CH-4	4.09	m	72.0
	CH_2_-5,5	1.87	ddd: 12.6; 3.3; 2.2	48.3
		1.55	t: 12.2	48.3
	C-6			36.6
	CH_3_-7,8	1.23	s	27.9
	CH_3_-7,8	1.24	s	29.3
	CH_3_-9	2.15 *	d < 1 Hz	19.3
	CHO-10	10.09	s	
(β-d-glucosyl)	CH-1′	4.44	d: 7.8	102.6
	CH-2′	3.16	dd: 9.2; 7.8	75.2
	CH-3′	3.35	m	78.1
	CH-4′	3.28	m	78.0
	CH-5′	3.29	m	71.7
	CH_2_-6′,6′	3.67	m	62.7
		3.86	t: 11.2	
*α-Glucose* αGLC	CH-1	5.10 *	d: 3.7	94.0
	CH-2	3.35	dd: 9.6; 3.7	73.9
	CH-3	3.67	dd: 9.6; 9.0	74.9
	CH-4	3.30	m	71.9
	CH-5	3.77		73.0
	CH_2_-6, 6	3.78; 3.69		62.8
*β-Glucose* βGLC	CH-1	4.47	d: 7.8	98.3
	CH-2	3.12 *	dd: 9.2; 7.8	76.3
	CH-3	3.33		78.2
	CH-5	3.27		78.1
	CH_2_-6,6	3.85; 3.65		62.9

### 3.3. Comparison between Saffron Samples of Different Geographical Origin

**Figure 4:**
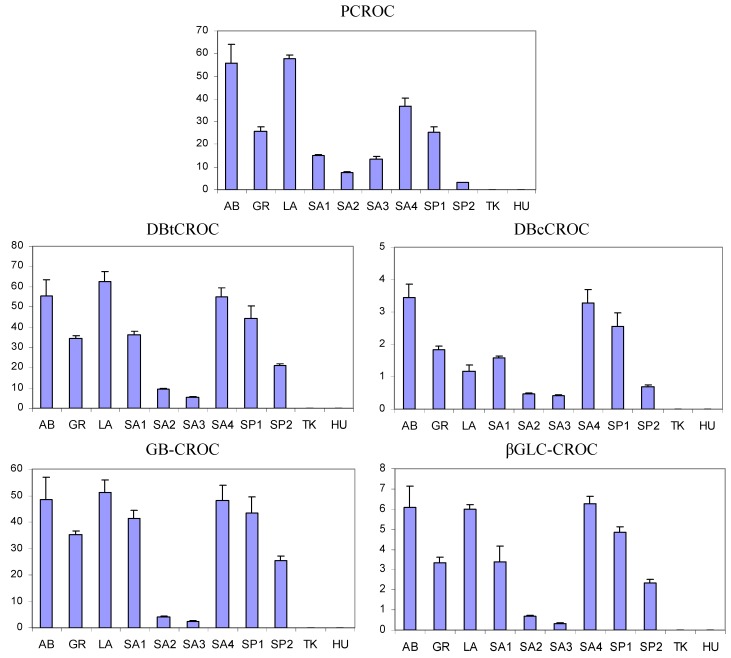
Histograms relative to the intensity of the selected metabolites (arbitrary units); see Table 1 and Table 2 for the abbreviations. Mean values and standard deviations for each metabolite are reported.

**Figure 5:**
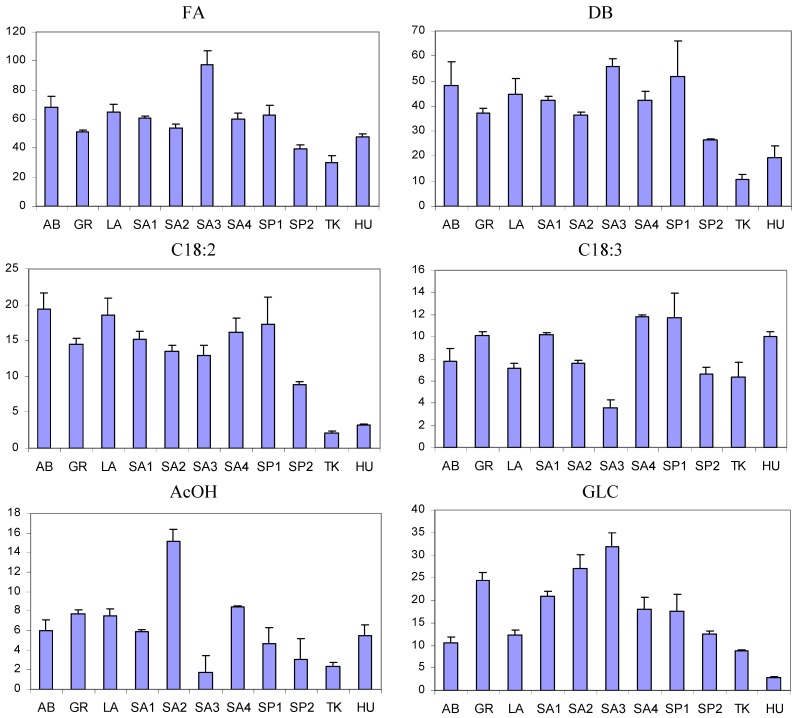
Histograms relative to the intensity of selected metabolites (arbitrary units); see Table 1 and Table 2 for abbreviations. Mean values and standard deviations for each metabolite are reported.

**Figure 6:**
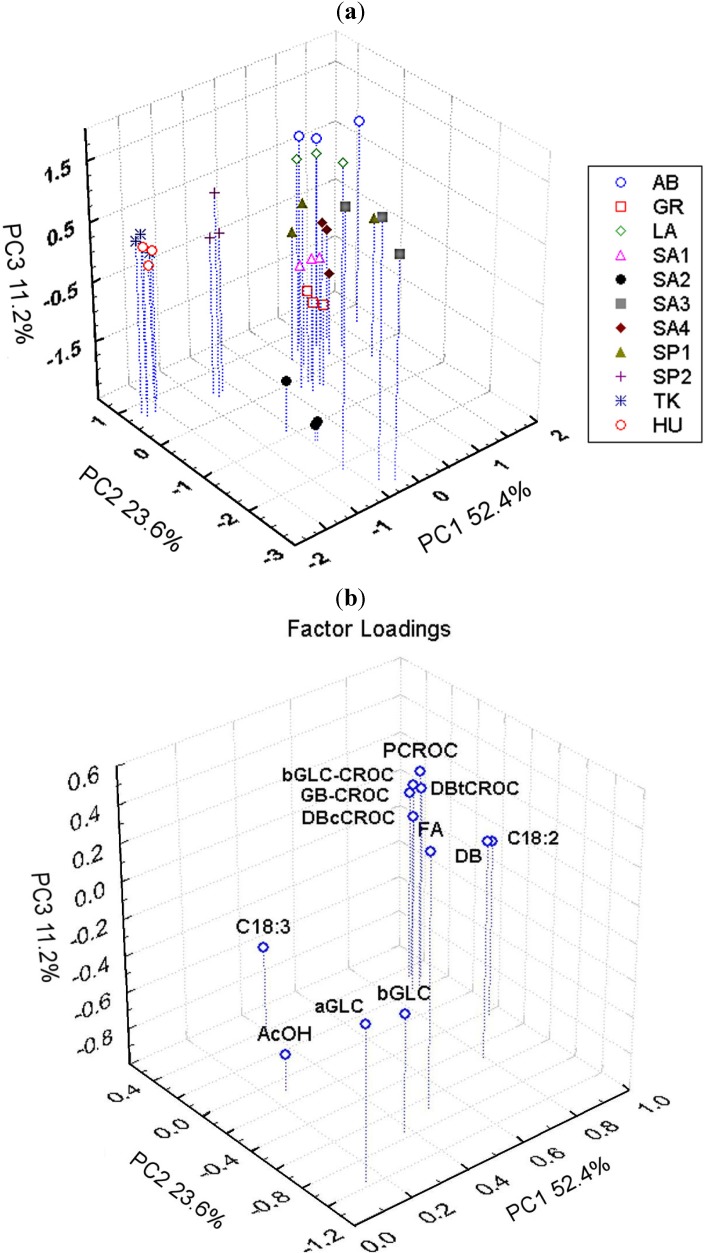
(**a**) PCA performed on 12 metabolite levels measured in saffron extracts of different geographical origin sample scores plot; (**b**) PCA plot of loadings.

The metabolic profiles of saffron extracts of different geographical origin and distinct production showed important dissimilarities, as evidenced in the histograms reported in Figure 4 and Figure 5 and in the PCA plots reported in Figure 6. In particular, as far as PCA is concerned, three components were evaluated to be significant according to an 11-fold cross-validation procedure, where, in each split, all the replicates of each sample were left out. The PCA applied to the integral of 12 selected ^1^H resonances shows that samples are grouped according to their geographical origin and specific production sites. The first three PCs account for 87.2% of the variability within the data, PC1 providing 52.4%, PC2 23.6% and PC3 11.2%.

The distribution of sample scores along the PC1 axis (the most important variability factor) seems to be reflected by the content of picrocrocin and crocetin esters (crocins), the main components of genuine saffron spice. In fact, saffron samples with the lowest PC1 scores are from Hungary (HU) and Turkey (TK), where these components were not detected, and they are well separated from all other samples. It is possible to hypothesize that HU and TK samples are not saffron samples. The samples with the highest PC1 scores were the certified AB (Abruzzi) PDO samples, commercial LA (Latium) ones and PDO samples SA4 (Sardinia) with the highest content of picrocrocin and crocins; see Figure 4. Other samples from Sardinia, Greece and Spain are in between these extremes.

The distribution of samples along PC2 and PC3 axes was due to the different content of other metabolites, such as glucose, AcOH and FA. FA and GLC are particularly abundant in the SA3 sample, which, in turn, is poor in AcOH, PCROC and crocins; see Figure 4, Figure 5 and Figure 6b. The samples from the same geographical area (Sardinia or Spain), but from different local producers were also separated in PCA plots, suggesting the importance of crop production technology together with environmental factors for the metabolite composition.

The analysis of histograms in Figure 4 showed that five variables due to picrocrocin and crocins were highly correlated. The correlation is also evidenced by the closeness of the PCA loadings of these five variables; see Figure 6b. The correlation between DBtCROC, DBcCROC, GB-CROC and bGLC- CROC due to the aglycone and glycosidic moieties of crocins was expected, because they in fact belong to the same type of molecules, *i.e.*, glycosyl esters of crocetin. The correlation of DBtCROC, DBcCROC, GB-CROC, bGLC-CROC with picrocrocin is quite unexpected. This probably indicates the correlated metabolic pathways of biosynthesis of picrocrocin and crocins. The correlation between all *trans* crocetin and 13-*cis*-crocetin moieties of crocins is not perfect, as can be seen from the ratio DBtCROC/DBcCROC reported in Table 3. In the case of LA samples, this ratio is two or three times higher than in other samples.

The results of NMR analysis and PCA can be applied to the estimation of the quality of saffron samples. According to ISO specifications [13], there are four quality categories of spice, ranging mainly according to the content of picrocrocin and crocins measured by UV-Vis spectrophotometry. The first category, with the highest quality, is characterized by the highest contents of these specific components. The content of picrocrocin and crocins can be directly measured by NMR spectroscopy, and therefore, the NMR analysis is suitable for spice quality determination, as well. The samples analyzed in this study can be ranked according to picrocrocin and crocins content and, therefore, their quality. AB PDO samples, LA ones and PDO samples SA4 have higher quality than other samples. HU and TK samples where no picrocrocin or crocins were detected were probably adulterated.

**Table 3:** Medium DBtCROC/DBcCROC ratio.

Sample code	DBtCROC/DBcCROC
AB	16.0
GR	18.7
LA	54.2
SA1	22.8
SA2	19.8
SA3	12.8
SA4	17.3
SP1	17.3
SP2	30.2
TK	n.d.
HU	n.d.

n.d.: not determined.

## 4. Conclusions

An NMR-based approach is proposed to characterize saffron extracts of different origin after microwave-assisted extraction. Taking into account the higher variability of the content of the spice constituents, which is probably influenced by the environment and the crop production technology, a proper analytical method is important for monitoring the quality of the product. Metabolite profiling could be used by the producer, either to standardize the production in order to obtain the same product every year or to optimize the crop production conditions.
